# Enhanced Antimicrobial Activity of AgCu Nanoparticles: The Role of Particle Size and Alloy Composition

**DOI:** 10.3390/molecules29133027

**Published:** 2024-06-26

**Authors:** Yuping Le, Fang Zhou, Longlai Yang, Yan Zhu, Dequan Yang

**Affiliations:** 1 Shanghai Technical Institute of Electronics & Information, 3098 Wahong Road, Shanghai 201411, China; 2 NanoTeX Lab, Solmont Technology Wuxi Co., Ltd., 228 Linghu Blvd, Tian’an Tech Park, A1-602, Wuxi 214135, China; 3 Solmont Technology Inc., 1305 Blvd Lebanc, Laval, QC H7E 4N5, Canada

**Keywords:** AgCu nanoparticles, antibacterial efficacy, particles size effects, bimetal nanoalloys

## Abstract

AgCu bimetallic· nanoparticles (NPs) represent a novel class of inorganic, broad-spectrum antimicrobial agents that offer enhanced antimicrobial effectiveness and reduced cytotoxicity compared to conventional Ag NP antibacterial materials. This study examines the antimicrobial performance and structural characteristics of AgCu nanoparticles (NPs) synthesized via two distinct chemical reduction processes using PVP-PVA as stabilizers. Despite identical chemical elements and sphere-like shapes in both synthesis methods, the resulting AgCu nanoparticles exhibited significant differences in size and antimicrobial properties. Notably, AgCu NPs with smaller average particle sizes demonstrated weaker antimicrobial activity, as assessed by the minimum inhibitory concentration (MIC) measurement, contrary to conventional expectations. However, larger average particle-sized AgCu NPs showed superior antimicrobial effectiveness. High-resolution transmission electron microscopy analysis revealed that nearly all larger particle-sized nanoparticles were AgCu nanoalloys. In contrast, the smaller particle-sized samples consisted of both AgCu alloys and monometallic Ag and Cu NPs. The fraction of Ag ions (relative to the total silver amount) in the larger AgCu NPs was found to be around 9%, compared to only 5% in that of the smaller AgCu NPs. This indicates that the AgCu alloy content significantly contributes to enhanced antibacterial efficacy, as a higher AgCu content results in the increased release of Ag ions. These findings suggest that the enhanced antimicrobial efficacy of AgCu NPs is primarily attributed to their chemical composition and phase structures, rather than the size of the nanoparticles.

## 1. Introduction

Silver nanoparticles (Ag NPs) and their metal alloys have attracted considerable attention in recent years due to their exceptional antibacterial properties and potential applications in various fields, such as healthcare [1,2], medical devices [3,4], and consumer products [5,6]. The growing prevalence of antibiotic-resistant bacteria has further stimulated interest in investigating alternative antibacterial agents, including silver nanoparticles and their alloys. Among the various silver metal alloys, silver–copper nanoparticles (AgCu NPs) have emerged as a promising candidate due to their enhanced antibacterial efficacy [7,8,9], synergistic effects [10,11,12], and lower cytotoxicity [8,10,13,14] compared to Ag nanoparticles alone.

The antibacterial performance of Ag NPs and their alloys is thought to be influenced by several factors such as particle size and shape [15,16,17,18], surface physicochemical properties [19,20,21,22], and the surrounding chemical environment [23,24,25]. It is widely accepted that smaller silver nanoparticles exhibit higher antibacterial activity [15,16,17,18]. This is attributed to the following two main reasons: First, smaller particles have a higher surface area-to-volume ratio, which facilitates the release of silver ions. Second, smaller particles can more easily penetrate bacterial cell walls, leading to cell death. As a result, the synthesis of small and stable silver nanoparticles and their alloys has become a primary focus in the field of antibacterial research.

However, the production of extremely small silver nanoparticles and their alloys presents challenges in terms of manufacturing feasibility and preservation stability. Therefore, it is essential to identify an optimal particle size that balances good antibacterial activity with ease of manufacture and storage stability. AgCu NPs have attracted particular interest not only for their enhanced antibacterial efficacy and synergistic effects but also for their potential in air-purification applications such as high-durability antibacterial filter coatings [26] and long-lasting antibacterial coatings [27,28].

The synthesis of AgCu nanoparticles (NPs) can be accomplished through various techniques, including chemical [9,29,30,31] and physical methods [32]. Our previous research has successfully demonstrated the synthesis of AgCu nanoalloy nanoparticles by directly adding copper ions to Ag NPs, facilitated by the reduction in Cu ions using polyvinyl alcohol-polyvinylpyrrolidone (PVA-PVP) as a catalyst at elevated temperatures. Additionally, we have managed to produce AgCu NPs at room temperature [12,30]. These methods have not only enhanced the antibacterial activity of Ag NPs but also improved their stability for practical applications.

Despite extensive research, the precise antibacterial mechanism of silver nanoparticles remains elusive [33,34,35]. However, it is generally believed to involve multiple factors such as the release of silver ions that interfere with bacterial DNA replication, the physical penetration of silver nanoparticles through bacterial cell walls, and the production of reactive oxygen species (ROS) that directly damage bacterial cells. The release of silver ions is considered a key aspect of the antibacterial action, as these ions can bind to bacterial DNA, preventing its replication and ultimately leading to cell death. Additionally, the small size of silver nanoparticles allows them to penetrate the bacterial cell wall, causing physical damage and disrupting cellular processes. Furthermore, the generation of ROS by silver nanoparticles can cause oxidative stress, damaging bacterial cell components and contributing to their demise. While these mechanisms are widely accepted, the complex interplay between them, and the specific contributions of each factor to the overall antibacterial efficacy of silver nanoparticles, remain the subject of ongoing research. Understanding these mechanisms in greater detail could lead to the development of more effective and targeted antibacterial treatments using silver nanoparticles and their alloys.

In this study, we aim to compare the antibacterial performance of AgCu nanoalloys prepared by two different chemical methods and investigate the relationship between particle size and antibacterial efficacy. Through high-resolution transmission electron microscopy (HR-TEM) analysis and antibacterial performance testing, we seek to shed light on the factors influencing the antibacterial properties of AgCu nanoparticles and provide insights into the underlying mechanisms. Our findings suggest that antibacterial performance is influenced not just by particle size, but also by particle structure, which regulates the AgCu nanoalloy content in the nanoparticles under identical chemical conditions. This research contributes to the understanding of the antibacterial behavior of AgCu nanoparticles and highlights the importance of tailoring particle structure and composition for enhanced antibacterial applications.

## 2. Results

**Figure 1:**
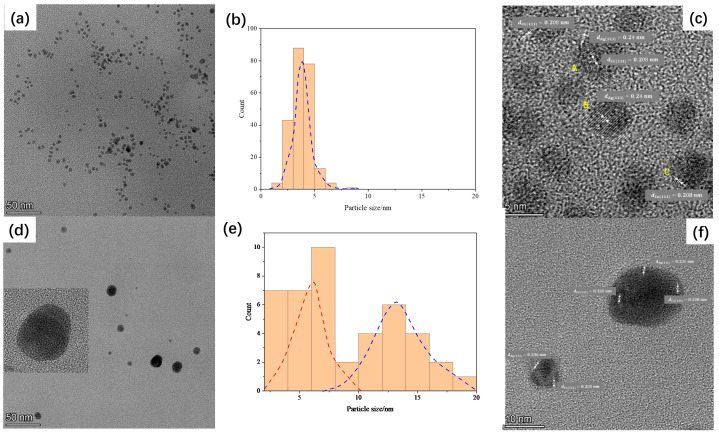
TEM microphotographs and their particle size distributions of sample AgCu-1# (**a**,**b**); and sample AgCu-2# (**d**,**e**). HR-TEM images of the sample AgCu-1# (**c**); and the sample AgCu-2# (**f**). The dotted lines in Figures (**b**,**e**) represent the fitted particle size distribution. A, B, and C correspond to AgCu alloy, Ag nanoparticles (NPs), and Cu nanoparticles (NPs), respectively.

Figure 1 shows TEM images of two types of AgCu NPs (AgCu-1# and AgCu-2# samples). The images reveal obvious differences between the two types of NPs, in both the particle morphological structure and size distribution, although both exhibit a spherical-like shape or polyhedron structure.

The TEM image of the AgCu-1# sample (Figure 1a) reveals a relatively uniform particle size distribution, with particle sizes within 10 nm and no obvious particle aggregation. The average particle size is approximately 4 nm (Figure 1b). In contrast, the particles of the AgCu-2# sample were significantly larger and exhibited a wider size distribution (Figure 1d,f), with most particles within 30 nm. The typical size distribution, shown in Figure 1e, can be summarized as two clusters: one centered at ~6 nm and another at ~13 nm for the AgCu-2# sample. For ease of comparison, we have included TEM images of typical Ag nanoparticles and their size distribution under similar conditions in Figure S1 of the Supplementary Materials. As illustrated in the figure, the average size of the Ag nanoparticles is approximately 5 nm, with all observed silver nanoparticles being within 10 nm.

High-resolution TEM (HR-TEM) observations, as shown in Figure 1c,f, revealed that the AgCu-1# particle sample contained not only an AgCu nanoalloy but also monometallic Ag and Cu nanoparticles (NPs). However, for the AgCu-2# sample, high-resolution TEM only observed the presence of bimetallic AgCu nanoalloy particles, with no monometallic Ag or Cu particles detected (Figure 1f).

**Figure 2:**
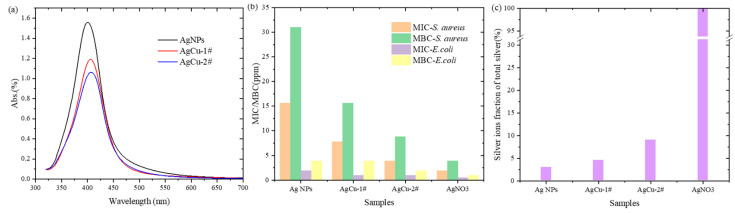
(**a**) UV–Vis spectra of AgCu samples and Ag NPs; (**b**) antibacterial efficacy, MIC/MBC of two AgCu samples and Ag NPs; and (**c**) Ag ions fraction in Ag and AgCu dispersions, measured by ICP-MS.

Figure 2a shows UV–Vis spectra of two AgCu samples and Ag NPs with a 10 ppm Ag concentration. Ag surface plasmon resonance (SPR) can be observed in all samples. The peak of Ag SPR shifts from 400 nm for Ag NPs to 406 nm for AgCu-1# and 407 nm for AgCu-2#. The peak position of the two AgCu samples is shifted to longer wavelengths (red shift), indicating the formation of AgCu alloy [12,30]. Additionally, the peak intensity of SPR decreases from Ag to AgCu-1# and AgCu-2#, revealing that AgCu has larger particle sizes and/or more alloy formations in the AgCu-2# sample. The higher peak intensity of the AgCu-1# sample is due to either the smaller nanoparticle sizes or the presence of some Ag NPs in the sample compared to the AgCu-2# sample, which is consistent with the TEM data in Figure 1.

The antibacterial performance, evaluated through minimum inhibitory concentration (MIC) and minimum bactericidal concentration (MBC) measurements shown in Figure 2b, reveals that the AgCu-2# sample possesses higher antibacterial efficacy compared to the AgCu-1# sample. Two AgCu samples demonstrate improved antibacterial effectiveness over Ag nanoparticles alone. Surprisingly, the AgCu-2# sample, despite having a larger particle size, exhibits the best antibacterial performance. This outcome is unexpected, as, generally, larger nanoparticle sizes are associated with reduced antibacterial efficacy for Ag nanoparticles, as documented in most experimental studies [16,17,18].

To explore the cause of this unexpected phenomenon, we measured the concentration of Ag ion release (relative fraction of total Ag) from 100 ppm solutions of Ag and AgCu, as depicted in Figure 2c. The order of the Ag ion fraction, from highest to lowest, is as follows: AgNO_3_, AgCu-2#, AgCu-1#, and Ag nanoparticles (Ag NPs). Notably, the fraction of Ag ion release in AgCu-2# is higher than in AgCu-1#, even though the average particle size of AgCu-1# is significantly smaller than that of AgCu-2#.

**Figure 3:**
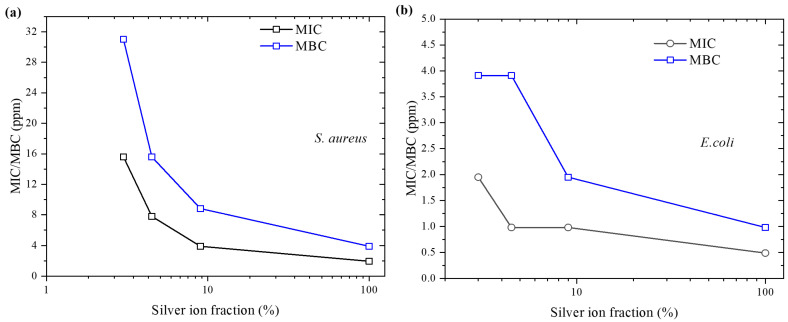
The effect of the silver ion release fraction (related to total silver) on antibacterial efficacy (MIC/MBC) against: (**a**) *S. aureus*; and (**b**) *E. coli.*

This suggests a correlation between the Ag ion fraction and antibacterial efficacy, with higher Ag ion concentrations resulting in better antibacterial performance. Consequently, the antibacterial efficacy of the AgCu samples, regardless of their average particle sizes, is directly related to Ag ion release. Figure 3 demonstrates that the MIC/MBC decreases with the increase in the Ag ion release concentration for the two strains.

## 3. Discussions

Both the surface morphology and particle distribution observed in the TEM images in Figure 1 suggest that AgCu-1# consists of a mixture of AgCu bimetallic alloy, monometallic Ag particles, and monometallic Cu particles. In contrast, AgCu-2# appears to be composed entirely of an AgCu bimetallic alloy within the observable region. Individual particles exhibit various atomic arrangements (labeled in Figure 1c), including structures with multiple domains (Figure 1f). This diversity in atomic configurations indicates the formation of a bimetallic alloy. HR-TEM images in Figure 1c,f reveal interplanar spacings of approximately 0.2 nm, corresponding to Cu(111) planes, and around 0.24 nm for Ag(111) planes, which are consistent with values reported in the literature [36,37,38,39]. Interestingly, the coexistence of AgCu nanoalloy, Ag NPs, and Cu NPs in the AgCu-1# sample has also been recently observed in an AgCu-PVA composite antibacterial coating, which was prepared by heating a mixture of Ag and Cu in a PVA matrix [27]. The observation of a single AgCu bimetallic alloy in the AgCu-2# sample is consistent with our previous findings from both high-temperature synthesis (conducted under the same conditions as in this study) [12] and room-temperature synthesis [30]. In summary, TEM data indicates that there is more AgCu nanoalloy formation in AgCu-2# compared to AgCu-1#, while AgCu-1# has significantly smaller particles than AgCu-2#. The UV–Vis spectra of the samples shown in Figure 2a further support the data from the TEM in Figure 1, indicating the formation of AgCu nanoalloys and demonstrating differences in their surface physical chemistry.

As we previously proposed [12], the formation of the AgCu nanoalloy can enhance Ag ion release due to the creation of a “microelectrochemical element cell” by the AgCu bimetal. Based on the HR-TEM data presented in Figure 1c,f, the higher Ag ion fraction in the AgCu-2# sample can be attributed to the presence of more AgCu nanoalloys compared to that of the AgCu-1# sample.

The antibacterial efficacy of these samples, assessed by MIC/MBC, against both *S. aureus* and *E. coli* is clearly correlated with the release of silver ions, as shown in Figure 3. Notably, the MIC/MBC values depend on the fraction of released silver ions for both strains, although they exhibit different capabilities against the two strains. This indicates that the antibacterial mechanism of the AgCu samples is predominantly governed by the release of silver ions, regardless of whether they are Ag NPs, AgCu NPs, or AgNO_3_.

Additionally, the silver ion release concentration effect (e.g., the fraction of Ag ions relative to the total Ag) exhibits a stronger correlation with the *S. aureus* strain compared to *E. coli*, as is evident in Figure 2b. Indeed, AgNO_3_ has the best antibacterial efficacy due to it having 100% Ag ions. The higher antibacterial efficacy of these silver-based samples against *E. coli* compared to *S. aureus* at a given silver concentration can be attributed to the fact that *S. aureus* has a thicker cell wall typical of Gram-positive bacteria, whereas *E. coli*, as a Gram-negative bacterium, has a thinner cell wall and an additional outer membrane. Ag NPs and Ag ions can more easily penetrate the outer membrane and cell wall of Gram-negative bacteria, allowing for more effective interaction with internal bacterial structures, leading to bacterial death [40,41]. The reason can also be attributed to the following factors: (i) Antibacterial Mechanisms: the antibacterial action of silver nanoparticles mainly involves binding to the bacterial cell wall and membrane, disrupting their structure, and interacting with internal proteins and DNA, interfering with their normal functions. Due to the structural differences between Gram-positive and Gram-negative bacteria, silver nanoparticles may more readily reach these targets in Gram-negative bacteria; (ii) Resistance Differences: *S. aureus* may exhibit higher resistance, including tolerance to silver. This resistance could be due to more complex biofilm formation or more effective mechanisms for expelling harmful substances; and (iii) Biofilm Formation: *S. aureus* can form dense biofilms, providing an additional protective layer for the bacteria, making it difficult for antimicrobial agents to penetrate. The protective effect of the biofilm may play a crucial role in resisting the effects of silver nanoparticles.

**Figure 4:**
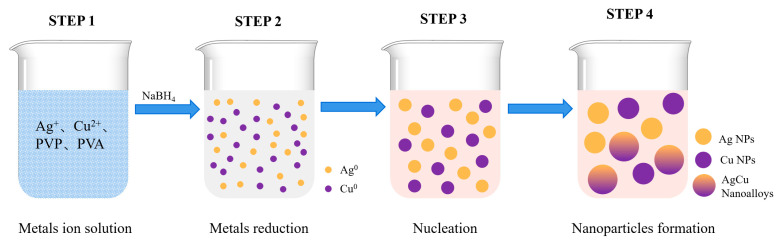
A schematic illustration of the formation of AgCu nanoparticles in the AgCu-1# sample.

A schematic of AgCu NPs formation in the AgCu-1# sample is illustrated in Figure 4. This is a very simple redox process. First, a solution containing silver and copper ions is prepared (STEP 1 in Figure 4). Immediately after adding an appropriate amount of sodium borohydride, the silver and copper ions are reduced to silver and copper atoms, respectively (STEP 2). These reduced silver and copper atoms will quickly nucleate (STEP 3) into three possible forms: copper clusters, silver clusters, and silver-copper clusters. Second, these nucleated clusters grow rapidly, but during the growth process, the presence of polyvinylpyrrolidone (PVP) and polyvinyl alcohol (PVA) prevents the small nanoparticles from clustering and growing further. Thus, three different nanoparticle structures can be rapidly formed (STEP 4). On the other hand, AgCu-2# particles are formed by the catalysis of silver nanoparticles and the reduction of copper ions by PVA-PVP [30]. This process first reduces copper ions to copper atoms, which are adsorbed on the surface of the silver nanoparticles and gradually form the AgCu structure. This structure helps to release silver ions from the silver nanoparticles, while the released silver ions are reduced by PVA-PVP and then combine with the AgCu structure. Finally, the AgCu structure gradually grows into larger particles, and the ungrown AgCu particles exist as smaller particles. This results in two AgCu nanoalloys of different sizes.

The two AgCu nanoparticle samples with the same PVP-PVA stabilizer, although having different average particle sizes, show a significantly lower relative content of AgCu alloys in the smaller particles (AgCu-1#). In contrast, the larger particles (AgCu-2#) are primarily composed of AgCu alloys. The formation of AgCu alloys can enhance or promote the release of silver ions, resulting in the lower relative amount of silver ions in AgCu-1# compared to AgCu-2#. As a result, the AgCu-2# sample exhibits superior antimicrobial efficacy despite its larger particle size, due to a higher concentration of released ions. The presence of AgCu alloys appears to be a critical factor in enhancing the release of silver ions and, consequently, the antimicrobial efficacy of these nanoparticles.

Although the strong effects of Ag NP shapes on antibacterial activity have been observed [42,43,44], with the antimicrobial activity estimated by the inhibition zone being in the order of spherical Ag NPs > disk Ag NPs > triangular plate Ag NPs [44], all the nanoparticles in our study, including AgCu, Ag, and Cu, possessed similar shapes, either spherical or polyhedral, as shown in the TEM images in Figure 1. Thus, we believe that the shape effects on the antibacterial efficacy of Ag, AgCu-1#, and AgCu-2# are minimal. Furthermore, the surface charges and chemical environments have a weak effect on antibacterial efficacy because the two AgCu samples have comparable chemical environments and pH values. Additionally, previous studies have indicated that the effects of Cu ions on antibacterial efficacy are minimal when compared to Ag [12,45]. Therefore, the higher antibacterial efficacy of the larger AgCu-2# nanoparticles can be attributed to their higher AgCu alloy content compared to the smaller AgCu-1# nanoparticles. Clearly, both the chemical composition and phase structures of AgCu NPs play critical roles in their antibacterial efficacy, as evidenced by the data from the two AgCu NP samples.

The preparation process of AgCu-2# is evidently environmentally friendly because it only involves the addition of Cu(NO_3_)_2_ without any additional reducing agents or stabilizers. This method presents a promising new approach for the preparation of new silver-based nanoalloys, such as AgFe and AgPt NPs, which have demonstrated excellent antibacterial and catalytic performance. We will discuss this further in future publications.

It should be mentioned that the two AgCu NPs samples exhibited high stability, as we observed minimal changes in both nanoparticle size and antibacterial efficiency after the samples underwent heat treatment at 60 °C for three months.

## 4. Materials and Methods

### 4.1. Materials and Samples Preparation

**Figure 5:**
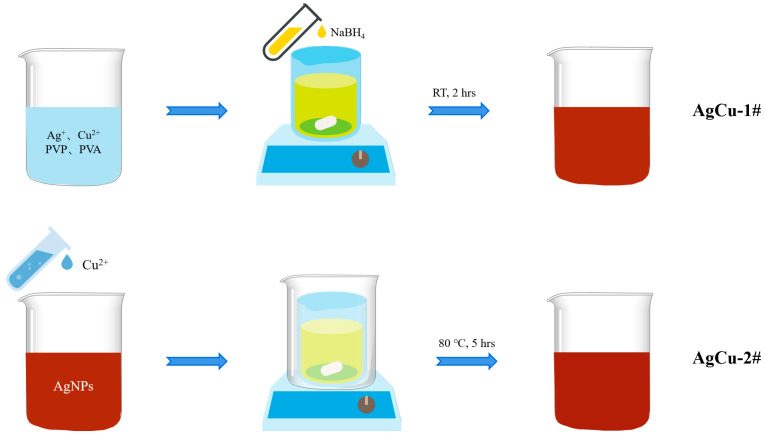
A schematic diagram of the sample preparation process for AgCu-1# and AgCu-2#. More detailed information on the preparation of sample AgCu-2# can be found in previous work [12].

A commercial aqueous dispersion of Ag NPs and AgCu NPs, both containing 1000 ppm Ag, with AgCu NPs having an additional 500 ppm Cu, was provided by Solmont Technologies Co., Ltd. (Wuxi, China). The nanoparticles were stabilized by polyvinyl pyrrolidone (PVP) and polyvinyl alcohol (PVA), as illustrated in Figure 5. The PVA had an average molecular weight of 100k (Solmont technologies, Wuxi, China), The PVP had a mass of 44–58k (g/mol) and was provided by Solmont Technologies (Wuxi, China). The use of both the PVA and PVP significantly improves the stability of the Ag NPs, extending their long-term aging time at both room temperature and at 60 °C, enabling their use in commercial applications. Cu(NO_3_)_2_ (Aladdin, AR grade, CAS No 10031-43-3) and Milli-Q water were used to synthesize the nanoalloys (NAs). All chemicals were used as received.

The AgCu nanoparticles (NPs) prepared by this method were labeled as AgCu-1#. Briefly, 0.78 g of AgNO_3_, 0.92 g of Cu(NO_3_)_2_·3H_2_O, 0.5 g of PVA, and 0.5 g of PVP were each added to 400 mL of water. After completely dissolving these components with stirring, 100 mL of a 1 M NaBH_4_ solution was added slowly and dropwise while stirring at room temperature. The reaction was allowed to proceed for 2 h to synthesize AgCu-1#. AgCu-2# was prepared by adding Cu ions to Ag NPs and then heating at 85 °C for 5 h, as illustrated in Figure 5. The detailed preparation can be found in our previous publication [12]. In short, AgCu-2# was prepared by taking 100 mL of the commercial Ag NPs in a 1000 ppm solution, adding 0.75 mL of a 1 M solution of Cu(NO_3_)_2_·3H_2_O, and allowing the reaction to proceed at 80 °C for 5 h. Both AgCu-1# and AgCu-2# had the same Cu and Ag concentrations and were stabilized by PVP-PVA for comparison purposes.

### 4.2. Characterization

UV–Vis spectra were recorded using a Yoke Instruments 723N spectrophotometer (Shanghai Yoke Instrument Co., Ltd., Shanghai, China). High-Resolution Transmission Electron Microscopy (HR-TEM) was carried out on an FEI Tecnai G2 F30 microscope (FEI, Basel, Switzerland).

MIC/MBC tests against *Staphylococcus aureus* (ATCC 25923) and *Escherichia coli* (ATCC 25922) were carried out by the broth dilution method [41]. Serial two-fold dilutions of AgCu NAs, in concentrations ranging from 500 mg/L to 0.244 mg/L, with adjusted bacterial concentrations (0.5 McFarland’s standard = 1.5 × 10^8^ CFU/mL, diluted 100 times with sterile saline to 1.5 × 10^6^ CFU/mL) were used to determine MIC in the nutrient broth: 100 μL of each dilution were placed in 96-well plates (Costar 96-well clear flat bottom plates, Corning, Corning, NY, USA) containing 100 μL of bacteria at 1.5 × 10^6^ CFU/mL and grown overnight at 37 °C. Visual observation was used to determine the MIC before and after incubation, and turbidity measurements [46] were used to confirm them.

To determine the minimal bactericidal concentration (MBC), diluted 100 μL aliquots of the samples were dispersed over trypticase soy agar (TSA) plates in the absence of bacterial growth. In the present study, the antibacterial effect of all the AgCu NAs and Ag+ (from AgNO_3_) were compared at the same Ag concentration. For the bacterial resistance evaluation of the NA/NPs, the samples were repeatedly incubated using 96-well plates, followed by MIC tests in new 96-well plates; this was conducted for three incubations. Each assay was repeated at least 3 times. Then, specific MIC and MBC values were determined.

### 4.3. ICP-MS Analysis

The measurement of Ag+ release was carried out using the following procedure: The samples were centrifuged at 12,000 rpm for 30 min. To each 1 mL of the resulting supernatant, 4 mL of 10% HNO_3_ were added to acidify the solution in preparation for analysis [47,48]. The concentrations of Ag+ in the solution were determined using inductively coupled plasma mass spectrometry (ICP-MS, Agilent 8800, Agilent, Santa Clara, CA, USA). Metal ions were detected three times for each sample, and the results from all repeated tests were reported as the mean ± standard deviation (SD).

## 5. Conclusions

Two types of AgCu nanoparticles with sphere-like shapes, synthesized using different chemical methods but with same chemical elements., were evaluated using TEM and antimicrobial performance measurements. The results showed that both types of AgCu nanoparticles demonstrated improved antimicrobial efficacy compared to Ag nanoparticles. However, the antimicrobial efficacy of smaller AgCu particles (with an average size of about 4 nm) was significantly lower than that of larger particles (with an average size of 14 nm), which contradicts typical findings. High-resolution TEM results revealed that the larger AgCu particles primarily consist of AgCu nanoalloys, while the smaller AgCu particles are a mixture of AgCu nanoalloys, Ag, and Cu monolithic nanoparticles. For the larger AgCu NPs (AgCu-2#), the higher proportion of the AgCu alloy enables the release of a greater quantity of silver ions. This is consistent with the fact that silver ion release is the primary factor in antimicrobial efficacy. Therefore, the size of AgCu nanoparticles does not necessarily directly determine their antimicrobial performance. Instead, the silver ion release capacity, which is governed by their chemical composition and specific structure (e.g., AgCu nanoalloy content in AgCu NPs), is the key factor in determining antimicrobial efficacy.

## Data Availability

Data are contained within the article and Supplementary Materials.

## Supplementary Materials

The supporting information can be downloaded at: https://www.mdpi.com/article/10.3390/molecules29133027/s1, Figure S1. (a) TEM image of Ag nanoparticles and (b) its size distribution.
