# Supplementary material for: Enhanced Antimicrobial Activity of AgCu Nanoparticles: The Role of Particle Size and Alloy Composition
Source: Molecules. 2024 Jun 26;29(13):3027. doi: 10.3390/molecules29133027 (PMC11242933; doi:10.3390/molecules29133027)
Supplement: Supplementary file 1 [file molecules-29-03027-s001.zip › molecules-2998416-supplementary.pdf]

Supplementary Materials

Enhanced Antimicrobial Activity of AgCu Nanoparticles: The Role of Particle Size and Alloy Composition

Yuping Le [1], Fang Zhou [2,†], Longlai Yang [2], Yan Zhu [1,2,*] and Dequan Yang [2,3,*]

[1] Shanghai Technical Institute of Electronics & Information, 3098 Wahong Road, Shanghai 201411, China; leyuping1@163.com
[2] NanoTe*X* Lab, Solmont Technology Wuxi Co., Ltd., 228 Linghu Blvd, Tian'an Tech Park, A1-602, Wuxi 214135, China; florazhou@g.ucla.edu (F.Z.); longlai.yang@solmontech.com (L.Y.)
[3] Solmont Technology Inc., 1305 Blvd Lebanc, Laval, QC H7E 4N5, Canada
* Correspondence: zhuyan@stiei.edu.cn (Y.Z.); dequan.yang@gmail.com (D.Y.)
† Current address: Department of Chemical and Biomolecular Engineering, University of California, Angeles, CA 90095, USA.

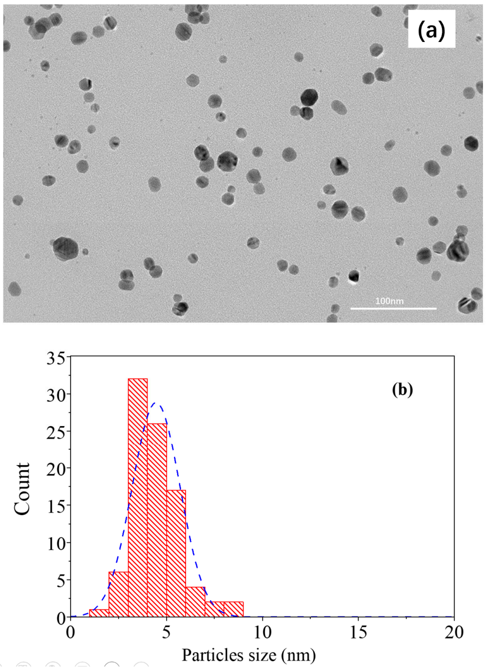

Figure S1. (a) TEM image of Ag nanoparticles and (b) its size distribution. The dot line is fitted size distribution.
